# Degradation of textile dyes using immobilized lignin peroxidase-like metalloporphines under mild experimental conditions

**DOI:** 10.1186/1752-153X-6-161

**Published:** 2012-12-20

**Authors:** Paolo Zucca, Antonio Rescigno, Manuela Pintus, Andrea C Rinaldi, Enrico Sanjust

**Affiliations:** 1 Dipartimento di Scienze Biomediche, Università di Cagliari, Cittadella Universitaria, 09042, Monserrato, CA, Italy; 2 Consorzio UNO (University of Oristano Consortium), Oristano, Italy

**Keywords:** Biomimetic, Dye, Lignin-peroxidase, Porphine, Porphyrin

## Abstract

**Background:**

Synthetic dyes represent a broad and heterogeneous class of durable pollutants, that are released in large amounts by the textile industry. The ability of two immobilized metalloporphines (structurally emulating the ligninolytic peroxidases) to bleach six chosen dyes (alizarin red S, phenosafranine, xylenol orange, methylene blue, methyl green, and methyl orange) was compared to enzymatic catalysts. To achieve a green and sustainable process, very mild conditions were chosen.

**Results:**

IPS/MnTSPP was the most promising biomimetic catalyst as it was able to effectively and quickly bleach all tested dyes. Biomimetic catalysis was fully characterized: maximum activity was centered at neutral pH, in the absence of any organic solvent, using hydrogen peroxide as the oxidant. The immobilized metalloporphine kept a large part of its activity during multi-cycle use; however, well-known redox mediators were not able to increase its catalytic activity. IPS/MnTSPP was also more promising for use in industrial applications than its enzymatic counterparts (lignin peroxidase, laccase, manganese peroxidase, and horseradish peroxidase).

**Conclusions:**

On the whole, the conditions were very mild (standard pressure, room temperature and neutral pH, using no organic solvents, and the most environmental-friendly oxidant) and a significant bleaching and partial mineralization of the dyes was achieved in approximately 1 h. Therefore, the process was consistent with large-scale applications. The biomimetic catalyst also had more promising features than the enzymatic catalysts.

## Background

Textile dyes are a broad and heterogeneous class of molecules used in many technological fields (i.e., textile and paper production, food technology, and hair coloring) [1]. Many chemical classes of synthetic dyes are frequently employed on an industrial scale; azo dyes, triarylmethanes, phenothiazine, and anthraquinones are among the most widespread [2,3].

Because the goal of the dyeing process is color durability, textile dyes of all chemical classes are commonly highly resistant to both chemical and physical degradation. Accordingly, textile dyes represent a serious environmental concern because of their chemical inertness, high annual production (over 10,000 tons per year [1]), toxicity/carcinogenicity [4,5], and the large amount of dyes released in industrial wastewaters (up to 50% of total process intake [6]).

The European Directive 2002/61/EC forbids the use of some products (in particular some azo dyes). However, these restrictions only marginally solve the environmental problem because of the large volume of other dyes discharged every year.

Several physical, chemical and enzymatic approaches have been proposed to remove dyes from industrial wastewaters [7-16] that require long reaction times, extreme operational conditions, and high costs [1]. Accordingly, the development of a clean and environmentally friendly process for removing dyes is a challenging issue, far from being solved.

Redox-active metalloporphines are efficient catalysts; in the presence of a proper oxidant [17-21], they are able to oxidize many substrates, including several textile dyes, under very mild conditions. We previously studied the ability of an immobilized manganese porphine to oxidize alizarin red S (ARS), an anthraquinone dye) [22], and phenosafranine (PNS), a phenylphenazinium dye [23], elucidating several aspects of the catalytic mechanism.

**Figure 1:**
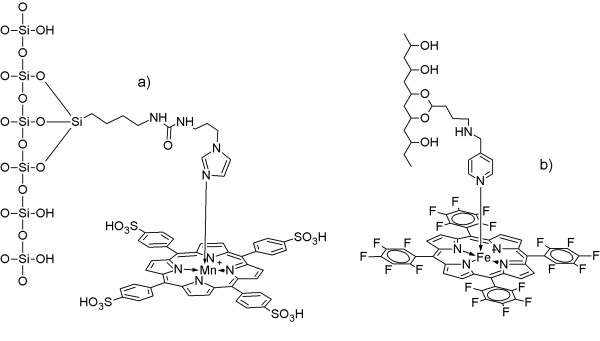
**Proposed structure of the catalysts IPS/MnTSPP (a) and PP-PVA/FeTFPP (b).** For more details, see: [24,25].

In this study, we compare the catalysts IPS/MnTSPP and PP-PVA/FeTFPP (Figure 1), each containing a metalloporphine (Mn- and Fe-, respectively) immobilized onto a solid support, thus emulating ligninolytic peroxidases [24]. We investigated the ability of these two catalysts to oxidize different chemical classes of textile dyes in the presence of hydrogen peroxide under mild conditions. We also report the results of these catalysts on ARS and PNS to allow a comprehensive comparison. All the dyes included in the study are reported in Table 1.

**Table 1:** The six textile dyes included in this study

**Dye**		**MW (g/mol)**	**λ**_**max**_**(nm)**	ε (mol * L^-1^* cm^**-1**^**)**
Alizarin Red S (ARS)		342.26	520	7200
Phenosafranine (PNS)		322.80	520	32000
Xylenol Orange (XO)		782.56	435	10500
Methylene Blue (MB)		319.86	660	79500
Methyl Green (MG)		472.51	632	55700
Methyl Orange (MO)		327.34	464	24800

## Results and discussion

### Comparison of IPS/MnTSPP and PP-PVA/FeTFPP

We previously developed two biomimetic catalysts, IPS/MnTSPP and PP-PVA/FeTFPP, which are able to oxidize several lignin-model compounds. IPS/MnTSPP strictly emulates a lignin peroxidase (LiP) active site, having an imidazole-grafted support that coordinates a Mn(III)-porphine [24]. This catalyst showed a LiP-like activity, whereas, unlike an enzyme such as manganese peroxidase (MnP), it was not able to use the Mn^2+^ ion as a redox mediator. Conversely, PP-PVA/FeTFPP was only “bioinspired” (i.e., only inspired by but not strictly emulating, a natural enzyme active site) because the metalloporphine was coordinated by a pyridine-grafted support [25] rather than imidazole, as in ligninolytic peroxidases. This catalyst showed both LiP-like and MnP-like catalytic activities.

These two catalysts were tested for their ability to decolorize several textile dyes in the presence and absence of Mn^2+^ using hydrogen peroxide as the oxidant in accordance with green chemistry principles [26]. The investigation employed very mild conditions (neutral pH and room temperature, using no organic solvents, and the most environmental-friendly oxidant) to serve as the basis for an environmentally sustainable textile wastewater treatment process.

The results of these tests are reported in Table 2. Both biomimetic catalysts easily degraded all tested dyes, showing wide substrate specificity. As previously reported [24], the catalytic behavior of IPS/MnTSPP was not affected by the presence of Mn(II), showing similar bleaching percentages in both the presence and absence of this ion. All six dyes were rapidly bleached under the conditions studied; more than with a removal in all cases higher than 50% of each dye (up to > 90% for PNS, MG, and MO) was removed in 1 h. In contrast, PP-PVA/FeTFPP was almost inactive in the absence of a suitable redox mediator. The conversion of all six dyes increased in the presence of Mn^2+^, leading to values similar on average to those observed for IPS/MnTSPP.

**Table 2:** Removal of the studied dyes in the presence of hydrogen peroxide and either IPS/MnTSPP or PP-PVA/FeTFPP, in both the presence and absence of Mn(II) as the redox mediator

**Mn(II)**	**Catalyst**	**Dyes removal % (1 h)**					
		**ARS**	**PNS**	**XO**	**MB**	**MG**	**MO**
No	IPS/MnTSPP	62 ± 1	92 ± 2	42 ± 4	96 ± 4	82 ± 5	87 ± 2
	PP-PVA/FeTFPP	n.d.	n.d.	n.d.	n.d.	n.d.	n.d.
Yes	IPS/MnTSPP	55 ± 1	88 ± 2	44 ± 2	91 ± 3	77 ± 4	85 ± 4
	PP-PVA/FeTFPP	48 ± 3	58 ± 2	55 ± 5	37 ± 1	48 ± 2	91 ± 2

The reaction mixtures contained 5 mg of catalyst in 1 mL of 25 mM buffer solution containing 8.8 mM hydrogen peroxide and a proper concentration of the selected dyes (0.29 mM ARS, 0.31 mM PNS, 2 mM XO, 0.15 mM MB, 1.5 mM MG, and 1.25 mM MO). Quantification of the removed dye was obtained photometrically, using the λ_max_ and molar extinction coefficients reported in Table 1. n.d.: not detectable. (*n* = 4).

Considering that textile wastewaters do not usually contain manganous ion and that it would therefore be necessary to add Mn^2+^, itself a heavy metal pollutant, to the reaction, IPS/MnTSPP was considered the most promising catalyst for future industrial applications Hence, the rest of study focused on IPS/MnTSPP.

**Figure 2:**
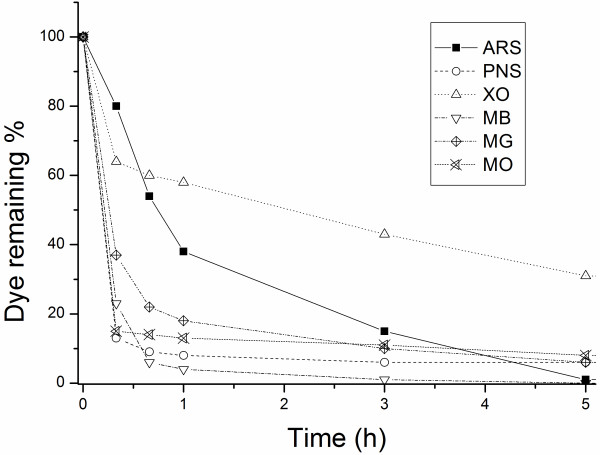
**Time dependence of dye removal in the presence of IPS/MnTSPP and hydrogen peroxide.** The reaction mixtures contained 5 mg of catalyst in 1 mL of 25 mM buffer solution containing 8.8 mM hydrogen peroxide and a proper concentration of the selected dyes (0.29 mM ARS, 0.31 mM PNS, 2 mM XO, 0.15 mM MB, 1.5 mM MG, and 1.25 mM MO). Quantification of the dyes was obtained photometrically, using the λ_max_ and molar extinction coefficients reported in Table 1.

The time dependence of dye bleaching by IPS/MnTSPP is shown in Figure 2. Note that most of the bleaching occurs in the first hour of the reaction, consistent with industrial timing needs.

### Characterization of bleaching by IPS/MnTSPP and H_2_O_2_

IPS/MnTSPP catalysis was strongly pH-dependent; an approximately neutral pH was optimal for all dyes tested except XO and PNS, which had optimal bleaching at pH 6 and 8, respectively (data not shown). However, XO and PNS were sufficiently bleached at pH 7 that IPS/MnTSPP remains a viable catalyst for environmentally friendly wastewater treatment.

An exhaustive kinetic modeling of this catalysis is beyond the scope of our work. However, the decolorization of all six dyes apparently followed saturation Michaelis-Menten-like kinetics, allowing the calculation of catalytic parameters (Table 3). Reported Michaelis constants (K_M_) showed that IPS/MnTSPP had significantly higher affinity for cationic dyes than anionic dyes (lower K_M_ for PNS and MB, higher K_M_ for ARS and XO). This lower affinity led to the lowest bleaching rate for XO and ARS (compare Figure 2). Notably, in all cases, K_M_ values were in the millimolar range and did not suffer from substrate inhibition. This can be a crucial issue for potential industrial applications because some bleaching approaches (i.e., enzymatic, *vide ultra*) are not able to work at these concentrations.

**Table 3:** Kinetic parameters measured for the bleaching of each dye by IPS/MnTSPP and hydrogen peroxide

**Sub-strate**	**Kinetic parameter**	**Dye**					
		**ARS**	**PNS**	**XO**	**MB**	**MG**	**MO**
**Reducing substrate**	K_*M*_ (mM)	2.11 ± 0.74	0.32 ± 0.04	1.33 ± 0.38	0.11 ± 0.02	0.72 ± 0.12	0.63 ± 0.05
	k_cat_ (min^-1^)	0.14 ± 0.03	2.2 ± 0.1	98 ± 14	19.0 ± 1.8	108 ± 9	67 ± 4
	k_cat_/K_*M*_ (mM^-1^ min^-1^)	0.066 ± 0.037	7.0 ± 1.2	74 ± 31	166 ± 52	150 ± 37	105 ± 16
**H**_**2**_**O**_**2**_	K_*M*_ (mM)	3.21 ± 0.12	3.0 ± 0.4	7.9 ± 1.9	2.4 ± 0.5	0.43 ± 0.08	7.4 ± 1.5
	k_cat_ (min^-1^)	0.13 ± 0.01	1.9 ± 0.1	78 ± 8.9	15.2 ± 1.3	159 ± 12	71 ± 8
	k_cat_/K_*M*_ (mM^-1^ min^-1^)	0.040 ± 0.004	0.63 ± 0.13	9.9 ± 3.6	6.4 ± 2.1	369 ± 100	9.6 ± 3.2

Data for ARS and PNS were previously reported and are added here to allow a full comparison [22,23] (*n* = 5).

To test the hypothesis of a one-electron abstraction mechanism as the first step of the bleaching process, the influence of several well-known redox mediators was also studied: 4-hydroxy-2,2,6,6-tetramethylpiperidine-1-oxyl (OH- TEMPO), *N*-hydroxyphthalimide (NPH), *N*-hydroxysuccinimide (NHS), and *N*-hydroxybenzotriazole (NHT). The results are summarized in Table 4. Only in the case of MG was an increase (three-fold) of the catalytic activity observed; in all the other cases, the redox-mediators mainly decreased IPS/MnTSPP catalysis (this quenching could be due to the lower reactivity of the oxygen-centered radical form of these mediators). Even in the best case, however, because the increase in catalytic activity was moderate, the industrial benefit of these redox mediators may not justify their environmental impact.

**Table 4:** Effect of well-known redox-mediators on the bleaching of the six tested dyes by IPS/MnTSPP

**Redox mediator**	**% Catalytic activity**					
	**ARS**	**PNS**	**XO**	**MB**	**MG**	**MO**
OH-TEMPO	98	45	277	56	336	16
NHT	142	46	162	38	299	121
NHS	141	89	146	90	313	122
NPH	103	32	74	56	340	57

Results are expressed by referring to a 100% reference activity in the absence of any mediator. Data for ARS and PNS were previously reported and are added here to allow a full comparison [22,23].

The catalyst was also investigated for its reuse potential. The results are reported in Table 5. On average, a rapid decrease of catalytic activity was observed in the first 2–3 cycles. Subsequently, activity was stable at approximately 25-30% of maximum for at least 8–10 cycles. A potential explanation for this pattern is the possibility that a fraction of some dyes remains tightly adsorbed on the catalyst even when the supernatant is completely decolorized. This adsorbed dye would likely reduce the catalytic performance by steric hindrance and/or electrostatic repulsion of incoming substrate molecules. Nevertheless, the catalyst keeps a significant part of its catalytic activity during multi-cycle employment. This is an important quality for large-scale applications.

**Table 5:** Multi-cycle activity of IPS/MnTSPP

**Catalytic cycle**	**% Catalytic activity**					
	**ARS**	**PNS**	**XO**	**MB**	**MG**	**MO**
1	100	100	100	100	100	100
2	68	73	100	99	98	44
3	49	70	93	73	97	37
4	33	61	88	49	97	34
5	30	51	47	37	97	28
6	28	49	41	34	96	28
7	28	44	31	34	96	28
8	26	41	30	26	94	27
9	25	39	29	25	94	20
10	25	35	27	24	90	19

Catalytic activity of the IPS/MnTSPP adduct upon multicyclic use during the decolorization of the studied dyes (*n* = 3). Data for ARS and PNS were previously reported and are added here to allow a full comparison [22,23].

**Figure 3:**
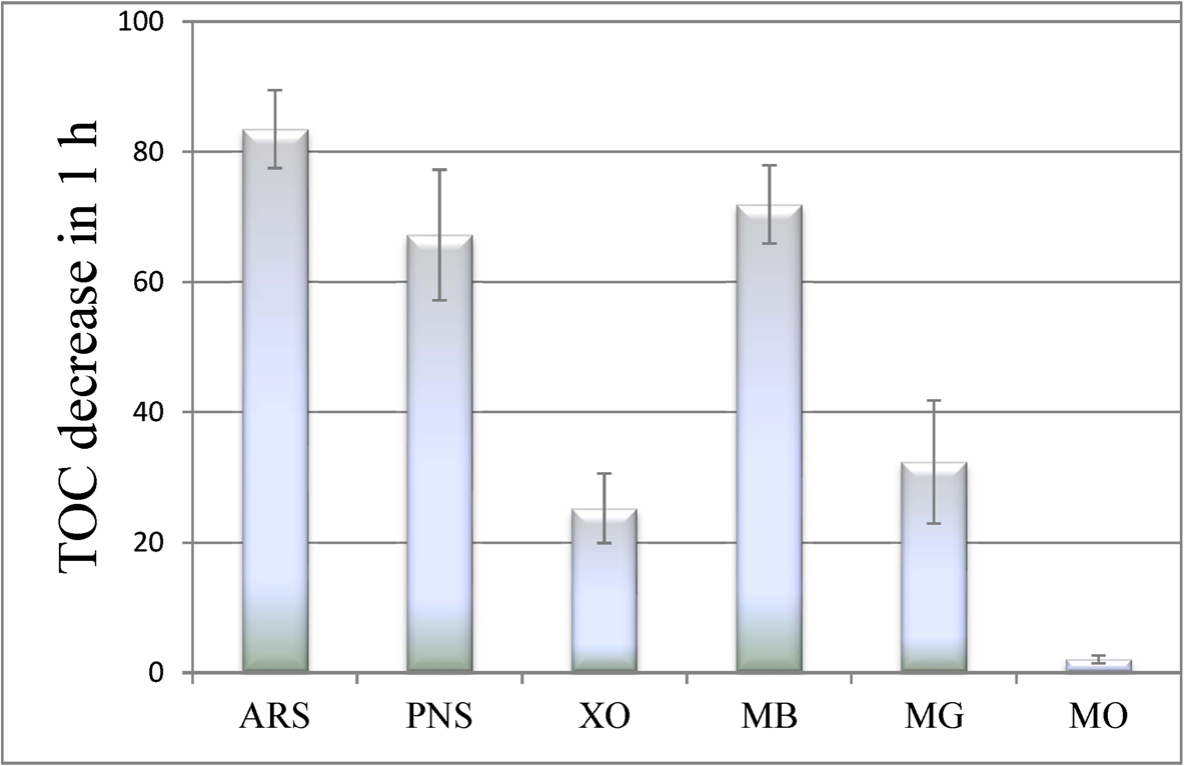
**Total organic carbon (TOC) decrease during the bleaching of the textile dyes by IPS/MnTSPP (*n*** **= 3).**

Significant removal of total organic carbon (TOC) was also observed during the bleaching by H_2_O_2_/IPS/MnTSPP, as shown in Figure 3. In the case of ARS, PNS, and MB, over 50% of TOC removal was observed in 24 hours, whereas lower decreases were recorded for XO and MG. Conversely, in the case of MO no TOC modification occurred during the catalysis. These results suggest that a decarboxylative degradation occurred for some of the dyes, whose TOC values decreased significantly during the bleaching experiments.

**Figure 4:**
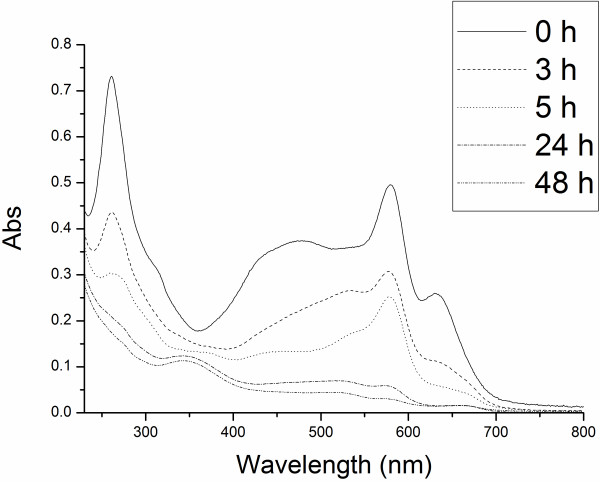
Changes in UV–vis spectra during the bleaching of a mix of the six studied dyes by IPS/MnTSPP.

IPS/MnTSPP was also tested for its ability to decolorize a complex mixture of all the six dyes, emulating realistic textile plant waste. The six dyes were each present in the saturating concentrations used above, leading to high impacting TOC (approximately 1580 mg/L). The changes in the UV–vis spectra (Figure 4) show a significant decolorization, although with a slower kinetics (approximately 5 hours for a 50% decrease of the main visible peak). However, the feasibility of using this treatment in an industrial process was confirmed by a significant TOC decrease (approximately 20%) in the same time frame.

### Comparison with enzymatic bleaching

A full comparison with the enzymatic bleaching of the dyes was also performed. The ligninolytic enzymes tested were laccase (LC), lignin peroxidase, and the non-ligninolytic enzyme horseradish peroxidase (HRP). In addition, Mn(III) was also used as oxidizing agent, mimicking manganese peroxidase action [27]. These biological catalysts suffered from significant substrate inhibition, preventing their use at the dye concentrations used for IPS/MnTSPP. Usually, concentrations of dyes at least of one order of magnitude lower were necessary. These enzymes also suffer from H_2_O_2_ inhibition, needing lower concentrations of oxidizing substrate. Accordingly, under the same conditions, it is not surprising that enzymatic bleaching efficiency was significantly lower than IPS/MnTSPP bleaching efficiency (<1% dye removal in all cases). Enzymatic bleaching activity, under the conditions studied, is reported in Table 6, allowing a qualitative analysis of substrate specificity only.

**Table 6:** Enzymatic bleaching of the dyes

**Dye**	**LC**	**LC + H**_**2**_**O**_**2**_	**HRP**	**LiP**	**Mn**^**3+**^
**ARS**	**+**	**++**	**+/−**	**+/−**	**+**
**PNS**	**-**	**-**	**-**	**+/−**	**-**
**XO**	**+**	**+**	**+**	**-**	**+**
**MB**	**-**	**+/−**	**+/−**	**+/−**	**-**
**MG**	**+**	**+**	**++**	**+/−**	**++**
**MO**	**+**	**+/−**	**+**	**+/−**	**+**

In the conditions tested, ligninolytic enzymes present a narrower substrate specificity towards the dyes included in this study than IPS/MnTSPP. Legend: LC laccase, HRP horseradish peroxidase, LiP lignin peroxidase, ++ good substrate (complete bleaching < 1 h), + substrate, +/− weak substrate (bleaching < 10% in 24 h), - no substrate.

None of the examined enzymes were able to bleach all the studied dyes. For instance, PNS is resistant to LC, HRP, and Mn^3^+, whereas only a partial degradation with concomitant irreversible enzyme inactivation is observed with LiP. MB underwent only partial oxidation with the enzymatic catalysts.

IPS/MnTSPP is able to effectively bleach all the dyes studied, as described above. This feature, combined with its ability to oxidize relatively high concentration of dye (in the millimolar range), and with its partial multi-cycling, may make IPS/MnTSPP catalysis a feasible alternative for industrial treatment of textile wastewaters.

## Experimental section

### Chemicals and instrumentation

All reagents were the best commercial grade available and were used without further purification. In particular, silica gel 100 came from Fluka (cat. No. 60746), and MnTSPP and FeTFPP were purchased from Sigma-Aldrich (cat. No. 441813 and 252913, respectively), PVA from Aldrich (cat. No. 363138), glutaraldehyde from Fluka (cat. no. 49629), horseradish peroxidase (E.C. 1.11.1.7) and lignin peroxidase (E.C. 1.11.1.14) were from Sigma-Aldrich (cat No. P-6782 and 42603, respectively). Laccase from *Pleurotus sajor-caju* was purified through two chromatographic steps (details in [28,29]).

Spectrophotometric measurements were carried out with an UltroSpec 2100pro (Amersham Bioscience). Concentrations of the substrates were also measured by UV-HPLC and GC-MS, using previously described methods [30,31].

### Preparation of IPS/MnTSPP

The complex IPS/MnTSPP was synthesized as previously described [24]. Briefly, 10 g plain silica gel was functionalized by overnight reaction at 80°C with 10 mmol 3-(1-imidazolyl)propylcarbamoyl-3’-aminopropyl-triethoxysilane solubilized in 10 mL dioxane, in the presence of 10 μL *N*-methylmorpholine acting as the catalyst. The activated silica (IPS) was washed consecutively with 0.5 M HCl, H_2_O, 0.1 M NaOH and H_2_O again. The wet silica was then carefully dried overnight in a vacuum oven.

The bound MnTSPP was quantified by the difference in absorption at 400 nm (the λ_max_ of the MnTSPP Soret band), as described in [24].

### Preparation of PP-PVA/FeTFPP

The complex PP-PVA/FeTFPP was synthesized as previously described [25]. Briefly, 1 g of aminopropyl crosslinked PVA (prepared as in [32]) was suspended in an excess of 0.05 M sodium acetate buffer pH 5, and treated with 0.1 mL 4-pyridinecarboxaldehyde and 0.5 g sodium cyanoborohydride. After 24 h of incubation at 25°C, the support was exhaustively washed with water, and dried at 50°C, to obtain PP-PVA. One-gram aliquots of PP-PVA were treated with 20 mg of FeTFPP, solubilized in 10 mL DMSO. The slurries were kept stirring in the dark for 24 h, washed exhaustively with DMSO, and carefully dried at 50°C.

Bound metalloporphine was quantified by difference through spectrophotometric measurement at 411 nm (ε_411_ = 115,000 M^-1^ cm^-1^ in DMSO) [25].

### Activity measurements

Routine measurements of the catalytic activity of immobilized metalloporphines were performed through spectrophotometric assays. The suspension contained 5 mg of catalyst in 1 mL of 25 mM buffer solution containing 8.8 mM hydrogen peroxide and a proper concentration of the selected dye. The routine analyses were performed using the following concentrations of dyes (chosen to be only slightly higher than respective K_M_, to better observe influences on catalytic activity): 0.29 mM ARS, 0.31 mM PNS, 2 mM XO, 0.15 mM MB, 1.5 mM MG, and 1.25 mM MO. During the kinetic experiments, wide ranges of substrate concentrations were studied (0.01-2 mM for the dyes, 0.1-40 mM for hydrogen peroxide).

The reaction mixture was then stirred at 25°C in the dark, and the reaction was monitored photometrically using the λ_max_ and molar extinction coefficients, reported in Table 1.

The actual hydrogen peroxide concentration was determined by KMnO_4_ titration.

When the reaction pH was not 7, the pH was corrected to 7 with 0.1 mL of potassium phosphate buffer 1 M (pH 7) before an absorbance measurement. UV–vis spectra in the range 230–700 nm were also recorded. Different McIlvaine buffers were used in the pH range 3–8.

When used, redox mediators (4-hydroxy-2,2,6,6-tetramethylpiperidine-1-oxyl OH- TEMPO; *N*-hydroxyphthalimide NPH; *N*-hydroxysuccinimide NHS; and *N*-hydroxybenzotriazole NHT) were added to the reaction medium at a final concentration of 1 mM. For the MnP-like assays, all the experiments were repeated as above in the presence of 1 mM MnSO_4_ and 50 mM malonic acid. During multi-cyclic runs, the catalysts were regenerated between consecutive cycles by repeated washings with water and 2-propanol and by drying at 50°C.

Total organic carbon determination was performed by automatic analyzer TOC 5000A (Shimadzu), equipped with Pt-Al_2_O_3_ oxidizing catalyst. The volume of injection was 25 μL. The calibration curve was obtained using a range of standard solutions of potassium phthalate (0–200 ppm).

Michaelis-Menten kinetic parameters were calculated by using R 2.5.1 software (*R Foundation for Statistical Computing*, Vienna).

### Enzymatic assays

When horseradish peroxidase (HRP) was used, up to 20 E.U. were present in a final volume of 1 mL of 25 mM buffer (pH 4, 5, 6, or 7) with 8.8 mM H_2_O_2_. Due to significant substrate inhibition, lower dyes concentrations were used: 119 μM ARS; 31 μM PNS; 200 μM XO; 15 μM MB; 150 μM MG; 65 μM MO.

The same conditions were adopted for the experiments with laccase (23 E.U. added); every experiment was performed in the presence and absence of H_2_O_2_.

In the case of lignin peroxidase (LiP), 0.2 E.U. were present in the final volume of 1 mL, with 0.176 mM H_2_O_2,_ due to strong substrate inhibition.

Moreover, to mimic manganese peroxidase (MnP) action, other experiments were conducted using Mn(III) as the putative oxidizing agent. One mM manganese triacetate was dissolved in 50 mM sodium malonate buffer, pH 6.5 [27], and the final mixture contained this solution along with dyes at the same concentrations, as mentioned above.

## Conclusion

IPS/MnTSPP has proved able to efficiently degrade all tested dyes, regardless of their chemical class, under very mild conditions: standard pressure, room temperature and neutral pH, using no organic solvents, and the most environmental-friendly oxidant. The yields and time requirements of the process were quite promising for a potential industrial application. Furthermore, a multi-cycle capability was described, even if only 30% of catalytic activity is retained for 8–10 cycles.

In comparison to enzymatic systems, the biomimetic catalyst was more efficient and had wider substrate specificity. Therefore, after proper optimization of the process, this catalyst is worth consideration as a possible medium/large scale application in textile wastewater treatment.

## Abbreviations

ARS: Alizarin Red S; FeTFPP: 5,10,15,20-tetrakis(pentafluorophenyl)porphine-iron(III) chloride; IPS: (3-Imidazolylpropylcarbamoyl)-3’-aminopropylsilica; HRP: Horseradish peroxidase; LC: Laccase; LiP: Lignin peroxidase; MB: Methylene Blue; MG: Methyl Green; MnTSPP: 5,10,15,20-tetrakis(4-sulfonato-phenyl)porphine-Mn(III) chloride; MO: Methyl Orange; MnP: Manganese peroxidase; PP-PVA: 4’-Pyridylmethyl-3-aminopropyl-functionalized; PNS: Phenosafranine; PVA: Crosslinked with glutaraldehyde; TOC: Total organic carbon; XO: Xylenol Orange.

## Competing interests

The authors declare that they have no competing interests.

## Authors’ contributions

PZ performed the main part of the experiments and drafted the manuscript. MP performed TOC and enzymatic assays. ACR, and AR helped analyzing the data and draft the manuscript. ES conceived the study, coordinated the study, and worked on data analysis and interpretation. All the authors read and approved the final manuscript.
